# Exploring Antimicrobial Resistance Profiles of *E. coli* Isolates in Dairy Cattle: A Baseline Study across Dairy Farms with Varied Husbandry Practices in Puerto Rico

**DOI:** 10.3390/microorganisms11122879

**Published:** 2023-11-28

**Authors:** Yadira Malavez, Sharon M. Nieves-Miranda, Paola N. Loperena Gonzalez, Adrian F. Padin-Lopez, Lingzi Xiaoli, Edward G. Dudley

**Affiliations:** 1 Department of Natural Sciences, University of Puerto Rico, Aguadilla, PR 00603, USA; smn5572@psu.edu (S.M.N.-M.); adrian.padin2@upr.edu (A.F.P.-L.); 2 Department of Biology, Industrial Biotechnology Program, University of Puerto Rico, Mayagüez, PR 00681, USA; 3 Department of Animal Sciences, Agricultural Experimental Station, University of Puerto Rico, Mayagüez, PR 00681, USA; 4 Department of Food Science, The Pennsylvania State University, University Park, PA 16802, USAegd100@psu.edu (E.G.D.); 5 *E. coli* Reference Center, The Pennsylvania State University, University Park, PA 16802, USA

**Keywords:** antimicrobial resistance, dairy farms, *E. coli*, Puerto Rico, whole-genome sequencing

## Abstract

Antimicrobial treatment in livestock can contribute to the emergence and spread of antimicrobial-resistant (AMR) microorganisms. Despite substantial surveillance of AMR bacteria in the continental United States, the prevalence of these AMR organisms in U.S. territories, such as Puerto Rico, remains understudied. The goals of this research included obtaining baseline data on the antimicrobial profile of *E. coli* isolates from Puerto Rico dairy farms with different husbandry practices. Seventy-nine fecal samples were collected from two types of conventional dairy farms: those that fed calves with tank milk and those that fed calves with waste milk. These samples were collected from the animals’ rectums, culture, and subsequently confirmed through biochemical tests. Out of these samples, 32 isolates were analyzed phenotypically and genotypically to elucidate their AMR profiles. The results underscore a discrepancy in the occurrence of antimicrobial resistance genes between calves and adult cattle. Notably, waste milk-fed calves exhibited a significantly higher prevalence of antibiotic-resistant *E. coli* when compared to their tank milk-fed counterparts. These disparities emphasize the need for more comprehensive investigations to determine causative factors. These results underscore the urgency of comprehensive strategies to raise awareness about how management practices influence antimicrobial resistance, shifting the focus from treatment to prevention.

## 1. Introduction

Antimicrobial drugs are essential for dairy farms to treat, prevent, and control diseases [1,2]. However, using antimicrobials in farm systems increases the development of antimicrobial resistance (AMR) in bacteria. AMR poses a concern since it can compromise human health by transmitting resistant pathogens from animals to humans [3]. For this reason, AMR has become a significant global public health concern that requires collaborative work between stakeholders, including public health agencies, health professionals, veterinary practitioners, and the general population, to mitigate its impacts. The World Health Organization (WHO) recommended an overall reduction in the use of all classes of medically important antimicrobials in food-producing animals and banned antibiotic usage for growth promotion [4]. Due to these recommendations in 2017, the Food and Drug Administration (FDA) prohibited using antibiotics for livestock without a veterinary prescription [5].

Farm management practices significantly affect product quality and animal health. Studies have reported that the overall health of cattle is enhanced on pasture diets requiring fewer antibiotics than animals fed with high-grain diets. Grain diet systems accelerate growth and fat accumulation, which are essential for mass production [6]. Calves are born with an underdeveloped rumen and initially rely on milk to supply nutrients for growth and overall health. Direct nursing and tank milk feeding stimulates the development of a healthy gut microbiota necessary for animal performance throughout its development [7,8]. Waste milk from cows undergoing antibiotic treatment for mastitis or other diseases is used to feed calves to mitigate economic losses because it is not fit for human consumption [9]. The consumption of milk from cows receiving antibiotic treatment may result in the emergence of antibiotic-resistant strains in calves [10].

About 62% of Puerto Rico’s dairy farms use waste milk to feed calves to mitigate the losses due to mastitis [11]. A 2014–2015 survey of 104 farms in the U.S. reported that about 40% of calves were fed with whole milk or waste milk, 35% with milk replacers, and 25% with a combination of the two. Also, about fourteen percent of U.S. farms use antibiotics as additives in the milk to feed calves [12]. Waste milk can exceed the tolerable levels of drug residues established by the FDA and increase antimicrobial resistance [13,14].

Studies have concluded that feeding calves with waste milk containing antimicrobial residues leads to the subsequent excretion of resistant bacteria [15]. Commensal organisms from non-human sources may transmit AMR genes to human pathogens, posing a significant public health threat [4]. Since 2019, about 4.95 million human deaths worldwide have been related to infections caused by antibiotic-resistant strains [16]; analyzing the sources of resistant strains to develop strategies to mitigate AMR is essential.

The dairy industry contributes 23% of the gross agricultural income in Puerto Rico [17]. In 2018, the dairy industry had an income of USD 172.2 million in sales [18]. Diseases in dairy cattle are a source of economic loss for dairy farms. For example, infections such as mastitis caused a financial loss of USD 10.3 million in Puerto Rico in 2014 [11]. Understanding AMR patterns in dairy cattle in Puerto Rico is essential to develop antimicrobial drug stewardship guidelines and management practices to prevent potential challenges that the dairy industry may encounter.

Fecal commensal bacteria, such as *Escherichia coli* (*E. coli*), have been widely used as indicator microorganisms for monitoring AMR [19]. *E. coli* is a rod-shaped Gram-negative bacterium that inhabits the gastrointestinal tract of warm-blooded animals such as cattle and humans [20]. Although most *E. coli* strains rarely cause illnesses, some are highly pathogenic and can cause intestinal diseases [21]. Diarrheagenic *E. coli* is classified by their virulence factors into pathotypes, which include the Shiga toxin-producing *E. coli* (STEC) [21]. Analysis of AMR in *E*. *coli* isolated from cattle is crucial to provide insights into the dissemination of AMR genes that can potentially impact human health.

This study compared two dairy farms with different husbandry practices in Puerto Rico. Our overarching hypothesis is that the antimicrobial profile of *E. coli* will be influenced by the feeding practice employed at each farm. This research aimed to obtain baseline data on the phenotypic and in silico antimicrobial resistance of the isolated *E. coli* strains.

## 2. Materials and Methods

### 2.1. Ethical Clearance

The University of Puerto Rico in Aguadilla Institutional Animal Care and Use Committee (IACUC) provided ethical clearance to the protocols used in the study (01-2017-IACUC-UPRAg). Permission was sought from farmers to retrieve samples from the cattle.

### 2.2. Samples and Processing

A total of 79 samples were collected from two commercial dairy farms in Puerto Rico between 2018 and 2019. The first farm fed calves (*n* = 13) with tank milk (TMF, Tank Milk Farm) and the second farm fed calves (*n* = 20) with waste milk (WMF, Waste Milk Farm). Cattle were fed in both farms with graze and grain (TMF *n* = 24, WMF *n* = 22).

The farm employees restrained cattle and calves for fecal sample collection. Samples were directly obtained from the rectum of each animal using sterile insemination gloves (cows) with 50% glycerol as a lubricant. After collection, gloves were turned inside out with a knot at the end and placed on ice [22]. Sterile cotton swabs were used to obtain calves’ fecal samples from the rectum of each animal [23]. After collection, swabs were stored in previously UV-sterilized resealable bags and placed on ice. All samples were transported to the laboratory within 2 h. For cattle samples, approximately one gram of each fecal sample was suspended in 9.0 mL of 0.09% NaCl (1:10) and mixed for 120 s in a stomacher paddle blender (Seward Medical, London, UK). Next, 1.0 mL of the mixed solution was transferred to Tryptic Soy Broth (TSB) (swab was placed into 10 mL of TSB for calf fecal samples. All sample tubes were incubated aerobically for 24 h at 37 °C.

### 2.3. E. coli Identification

**Figure 1:**
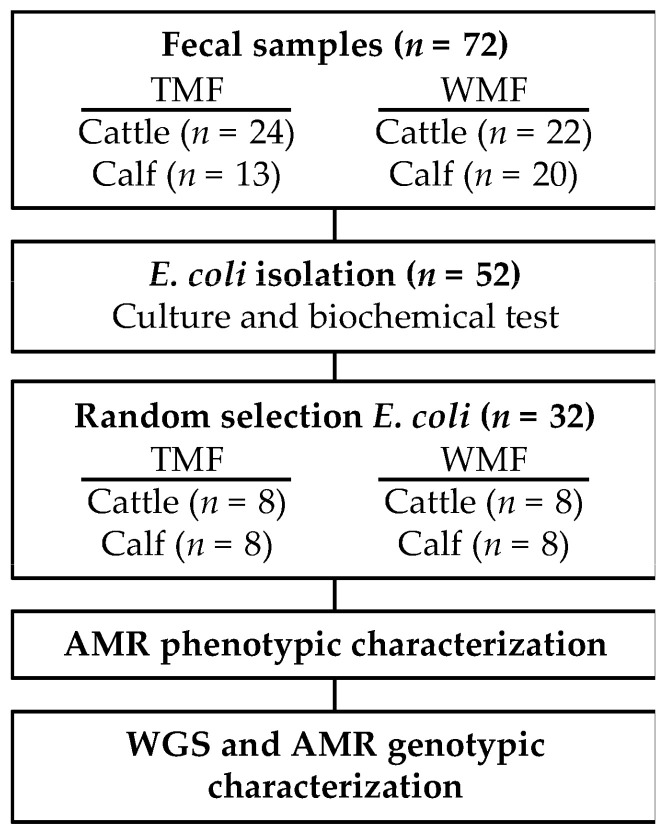
Study design summary. Isolates were collected from the tank milk farm (TMF) and waste milk farm (WMF). Abbreviations: AMR, antimicrobial resistance; TMF, tank milk farm; WMF, waste milk farm; WGS, whole genome sequencing.

*E. coli* was identified via culture and biochemical tests: TSB cultures were streaked onto Lactose MacConkey agar. Lactose fermenting colonies with a characteristic pink color were streaked on Eosin Methylene Blue plates, and the typical green-metallic colonies were subjected to the standard biochemical tests that included Sulfur-Indole-Motility (SINM) and Methyl Red/Voges-Proskauer (MR-VP). All media were incubated for 24 h at 37 °C. A total of 32 confirmed *E. coli* samples were chosen for phenotypic and genotypic characterization using whole-genome sequencing (WGS). These samples included 16 from cows (8 cows/farm) and 16 from calves (8 calves/farm), selected using a random number generator (Figure 1).

### 2.4. Antibiotic Susceptibility Testing

Antibiotic susceptibility testing was performed using the Kirby–Bauer disc diffusion method, as previously described [24]. Antibiotic resistance was determined according to the 2020 Clinical and Laboratory Standards Institute antimicrobial susceptibility tests standard breakpoints for *Enterobacteriaceae* [25]. The antimicrobial agents were amoxicillin/clavulanic acid (AMC, 20/10 µg), ampicillin (AMP, 10 µg), ciprofloxacin (CIP, 5 µg), gentamicin (GEN, 10 µg), kanamycin (KAN, 30 µg), sulfamethoxazole/trimethoprim (SMZ, 23.77 µg/1.25 µg), and tetracycline (TET, 30 µg) (Becton, Dickinson and Company, Franklin Lakes, NJ, USA).

### 2.5. DNA Extraction

DNA from a single colony was extracted using a Qiagen DNeasy Blood Kit (Qiagen, Hilden, Germany), and 1 mL was cultured in Luria–Bertani for 18 h at 37 °C. The purity of the extracted DNA was assessed with the Nanodrop Spectrophotometer N-1000 (Thermo Scientific, Madison, WI, USA) and the Invitrogen™ Qubit™ Fluorometer (Life Technologies, Marsiling, Singapore).

### 2.6. Whole Genome Sequencing and Bioinformatic Analyses

Genomic libraries of the *E. coli* isolates were created using the Nextera XT Library Preparation Kit (Juno Beach, FL, USA). WGS was performed using the MiSeq version 3 reagent kit with 600 cycles in an Illumina MiSeq benchtop sequencer (Illumina Inc., San Diego, CA, USA). Raw reads were uploaded to Galaxy Trakr [26]. Quality was assessed via FastQC v.0.72 [16]. Genomes were de novo assembled with SPAdes v.3.12.0 [27]. Assembly quality assessment with QUAST v5.0 [28], and the MLST was identified using Skesa v.0.0.3 [29]. Antimicrobial and serotyping of the *E. coli* isolates were determined using ResFinder v.3.2 [30] and ECTyper [31], respectively. Virulence was analyzed with Virulence Finder (v2.0) [32]. Default parameters were used for all software, unless specified.

The quality metrics used included an assembly was accepted if the average read quality score was >30, average coverage > 40X, de novo assembly (Mbp) 4.5–5.9, and number of contigs were <400.

## 3. Results

### Phenotypic and In Silico Antibiotic Resistance

**Figure 2:**
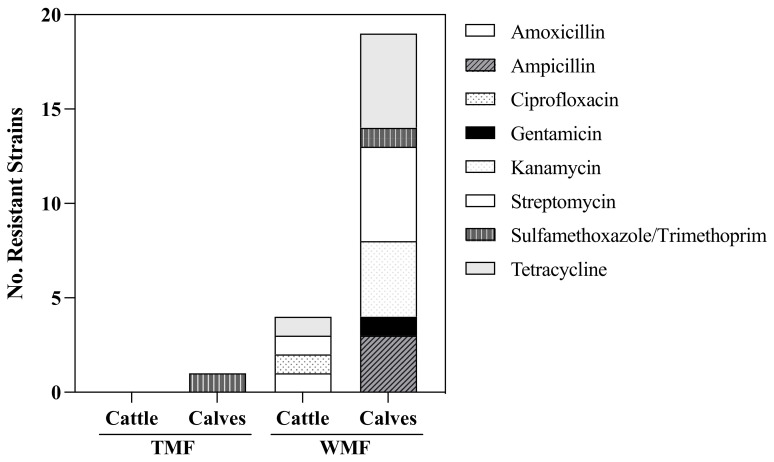
Phenotypic antibiotic resistance of 32 *E. coli* isolates from cattle and calves. Isolates were collected from the tank milk farm (TMF) and waste milk farm (WMF). The TMF fed calves with tank milk. The WMF fed calves with waste milk. Each set contains 8 *E. coli* isolates (*n* = 32).

A total of 79 fecal samples were obtained from two farms, and 52 presumptive *E. coli* isolates were identified (Figure 1). Thirty-two isolates were randomly selected for the AMR phenotypic characterization by Kirby Bauer disk diffusion test. Resistance to eight antibiotics was analyzed as an accepted protocol to evaluate resistance and sensitivity. The antimicrobials selection was made suitability for animal use. *E. coli* isolates from the WMF had higher resistance than the isolates from TMF (Figure 2). Three *E. coli* isolates from the waste milk-fed calves were resistant to ampicillin (38%), one to gentamicin (13%), four to kanamycin (50%), five to streptomycin (63%), one to sulfamethoxazole/trimethoprim (13%), and five to tetracycline (63%). One *E. coli* isolate (13%) from a WMF cow showed resistance to amoxicillin/clavulanic acid, ampicillin, kanamycin, streptomycin, and tetracycline. In contrast, one *E. coli* isolate (13%) from tank milk-fed calves showed resistance to sulfamethoxazole/trimethoprim. The eight *E. coli* isolates from TMF cattle were susceptible to all antibiotics.

The thirty-two *E. coli* selected isolates were subjected to whole-genome sequencing (WGS) followed by in silico antimicrobial-resistance prediction using ResFinder. This analysis identified the highest number of antimicrobial resistance genes in isolates from WMF for both cows (16%) and calves (60%) (Table 1). Isolates from WMF calves were predicted to have resistance genes against aminoglycosides (63%: *aac(3)-Ild, aadA1, aadA2, aph(3′)-Ia, aph(3″)-Ib, aph(6)-ld*), extended-spectrum beta-lactamases (38%: *blaTEM-1b*, *blaTEM-57, blaCTX-M1*), folate pathway antagonist (12%: *dfrA12*), phenicol (63%: *floR*), quinolones (38%: *qnrB19*), sulfonamides (63%: *sul1*, *sul2*, *sul3*), and tetracyclines (63%: *tetA*, *tetB*). Only one isolate from a WMF cow had resistance genes against aminoglycosides (*aph(3″)-Ib*, *aph(6)-ld*), phenicol (*floR*), and tetracycline (*tetA*). However, only one isolate from a TMF calf carried genes predicted to confer resistance to aminoglycoside (*aadA5*), folate pathway antagonist (*dfrA17*), and sulfonamide (*sul2*). Resistance to macrolides was predicted for all *E. coli* (*mdfA*) isolated from both farms. Multidrug resistance (MDR) was predicted in five isolates from WMF calves (63%), compared to one isolate in the TMF calves (13%). Among these MDR isolates, 50% had a predicted resistance to at least five classes of antibiotics and 13% had a predicted resistance to at least six antibiotics (Table 2).

MLST analysis found 25 different STs in the *E. coli* isolates. The most prevalent was ST8217 (*n* = 4) from TMF cattle, followed by the ST38 (*n* = 2, TMF cattle and calves), ST765 (*n* = 2, TMF and WMF calves), ST3580 (*n* = 2, WMF cattle), and ST10598 (*n* = 2, WMF cattle). The other STs comprise only one isolate each. The *E. coli* isolates were mainly assigned to the phylogenetic group B1 (*n* = 18), followed by A (*n* = 9), B2 (*n* = 1), D (*n* = 1), and E (*n* = 1). Virulence genes that permitted identification of pathotypes were observed in three isolates. UPR-0051 carried *stx1a* and *stx2a*, the genes encoding for two different Shiga toxins, classifying it as a Shiga toxin-producing *E. coli*, or STEC. Two other isolates (UPR-0053 and UPR-0085) carried *eae*, the gene encoding for the surface adherence protein intimin, classifying these as Enteropathogenic *E. coli*, or EPEC (Table 2).

Across all subsets, there was a 100% correlation between AMR phenotype and genotype for aminoglycosides and tetracycline, whereas there was between 87 and 96% correlation for beta-lactam, quinolone, and sulfonamide (Table 3). Additionally, no correlation for macrolide or phenicol could be calculated, as resistance to these drugs was not phenotypically tested for.

## 4. Discussion

This work investigated the antimicrobial profile of fecal *E. coli* isolated from cows and calves from two farms with different husbandry practices. Although many advances have been made in the U.S. to minimize the unnecessary use of antibiotics, at the time of this study, local farmers in Puerto Rico did not fully implement these regulations. To our knowledge, this study is the first baseline analysis of the antimicrobial resistance profile of fecal *E. coli* carried out in Puerto Rico dairy farms. Despite this study being limited in scope to perform statistical analysis, critical comparisons can be made to earlier literature, which will focus on our future efforts.

Interesting observations concerning AMR are worthy of follow-up studies. First, phenotypic and in silico AMR analyses showed that *E. coli* isolated from WMF calves had higher levels of AMR than TMF calves. Other studies have also reported that waste milk containing antibiotic residues increases the prevalence of antibiotic-resistant bacteria in calves [9,33,34,35]. Even low concentrations of antibiotics modulate gut microbiota and increase antimicrobial resistance in young animals [36].

*E. coli* isolates from WMF calves showed higher phenotypic resistance to tetracyclines (63%) and the aminoglycosides streptomycin (63%), kanamycin (50%), and gentamycin (13%) followed by resistance to the beta-lactam ampicillin (38%), and sulfonamides (13%). WGS predicted antimicrobial resistance genes against these antibiotics and resistance to extended-spectrum beta-lactamase, folate pathway, phenicol, quinolone, and macrolide antibiotics. The results are consistent with previous studies in the U.S. that have identified higher resistance to tetracyclines and aminoglycosides. A possible explanation for these findings is that tetracyclines, aminoglycosides, and macrolides antibiotics are approved to be used in the U.S. to treat mastitis, respiratory and reproductive diseases in dairy cattle along with other antibiotics such as fluoroquinolones, penicillin, and sulfonamides [37].

Our findings suggest that *E. coli* isolated from calves presents a higher antibiotic resistance level than cow isolates. This finding agrees with studies showing that AMR genes decline as animals age [38]. Also, all *E. coli* isolates had a predicted resistance to macrolides (*mdfA* gene) regardless of the farm management and age group. The *mdfA* is a chromosome-encoded gene that confers resistance to macrolide antibiotics commonly used in the U.S. to treat mastitis-associated *E. coli* infections [39]. The plasmid-encoded *mdfA* gene confers resistance to zwitterionic lipophilic or cationic compounds [40]. It has been related to cross-resistance to quaternary ammonium used as a post-milking disinfectant [41]. Further analysis is needed to determine macrolide usage and compare it to the experimental data to assess causation.

Furthermore, the *floR* gene was identified in five isolates from calves (63%) and one isolate from a cow (13%) from WMF but was not recognized in any of the isolates from the TMF. The *floR* gene confers resistance to florfenicol in *E. coli*. The florfenicol antibiotic was approved in the U.S. in 1996 for veterinary use in non-lactating dairy cattle to treat bovine respiratory disease. However, this drug is not labeled for use in female dairy cattle 20 months or older because the residues of the antibiotic can potentially pass into the milk, posing a risk to human health if consumed. This suggest that strains with florfenicol resistance may be selected when animals are treated at a young age. These findings could be explained by phenicol used as antimicrobial drugs for treating calf’s respiratory tract infections [42]. Since *E. coli* isolates with chromosomal and plasmid-encoded *floR* genes have been reported in diverse agriculture settings, the high prevalence of the *floR* gene in dairy cattle from Puerto Rico raises concerns about potential horizontal gene dissemination [43,44,45]. These findings raise the need for a more extensive study to assess the prevalence of *floR* and other AMR genes in *E. coli* isolates from dairy cattle in Puerto Rico.

The results of this study suggest that exposure of young animals to antimicrobial drugs may favor the development of antimicrobial resistance in the gut microbiota, including bacteria such as *E. coli*. The higher antimicrobial resistance observed in the *E. coli* isolates of WMF calves creates concern about the usage of antibiotics in dairy farms of Puerto Rico and the need to create awareness about the importance of nourishment to calves with milk without antibiotics. It is crucial to acknowledge that while antimicrobial drugs contribute to safeguarding cattle health, our results suggest that their extensive usage within farm systems is related to the increase in AMR in *E. coli* isolates. AMR impacts animal health and could potentially pose a risk to human health through the transmission of resistant pathogens [3]. Given that the antimicrobial resistances identified in this study are classified as critically important for human medicine by the WHO, it is crucial for future studies to examine the connections between the occurrence of AMR in human pathogens and antimicrobial usage on dairy farms in Puerto Rico [4].

Notably, few virulence genes were identified in our collection. Strain UPR-0051 carries genes for *stx1a* and *stx2a*, both associated with human pathogenic strains of STEC [46]. Interestingly, the sequence type of UPR-0051 (ST10760) is unique among strains categorized in the Enterobase database [9,47]. The UPR-0053 and UPR-0085 were both found to be ST765, carrying the gene for intimin (*eae*). In a previous study in Belgium [48], such strains were common among *eae*-positive *E. coli* isolates from cattle. Interestingly, most of those strains were also serotyped as O177:H11. These data collectively suggest that cattle may be a reservoir for ST765, *eae*-positive, O177:H11 *E. coli* and that these organisms have spread across a large geographic area (Table 2).

The commensal isolates examined in this research exhibit considerable genetic diversity, revealing 25 sequence types. This characteristic of extensive genomic plasticity within *E. coli* has been documented in various regions globally [48]. The adaptability of *E. coli* is pivotal in their capacity to proliferate within various environments, notably in the gastrointestinal tract of warm-blooded animals such as cattle. Gut commensal bacteria utilize virulence factors and antimicrobial resistance mechanisms crucial for their survival amidst challenges and in competing with other microorganisms [49]. Genomic plasticity is likely influenced by horizontal gene transfer, gene loss, point mutations, and DNA rearrangements, and the competitiveness of individual variants in diverse niches [50]. Our isolates predominantly fell into the two primary phylogroups, A and B1. This observation aligns with findings from other studies, which have consistently identified these as the primary phylogroups in food-producing animals [51,52]. As this study establishes the baseline study for evaluating *E. coli* in two Puerto Rican dairy farms, it is critical to expand the analysis to analyze samples from diverse farms across the island. Understanding the management practices employed throughout Puerto Rico is critical for comprehending the dynamics of antimicrobial resistance in *E. coli* with a broader comparison of virulence factors and clonal relationships among the *E. coli* isolates. This holistic approach aims to develop strategies that address the challenge of antimicrobial resistance, ultimately benefiting animal welfare and human health.

## 5. Conclusions

In summary, we have documented potential concerns surrounding AMR of *E. coli* isolates obtained from two dairy farms with different husbandry practices in Puerto Rico. These data highlight the importance of farm management and feeding of animals on the phenotypic and genetic profile of *E. coli* isolates. The results raise a need for more extensive studies to phenotypically and genotypically analyze AMR in the dairy cattle of Puerto Rico. Furthermore, it supports the need for holistic strategies for stewardship and training of farmers to create awareness of management practices’ influence on AMR to shift efforts from disease treatment to disease prevention, as has been carried out in other jurisdictions [5,53]. These strategies can contribute to reducing AMR’s burden on animals, aligned with the One Health approach that interconnects human, animal, and environmental health.

## Figures and Tables

**Table 1:** Prevalence of resistance genes in tank milk farm and waste milk farm.

Antibiotic Family	Gene	TMF		WMF		Total Number (%)
		Cattle (*n* = 8)	Calves (*n* = 8)	Cattle (*n* = 8)	Calves (*n* = 8)	
		No. Positive Strains				
Aminoglycoside	*aac(3)-Ild*	-*	-*	-*	1	16 (20)
	*aadA1*	-*	-*	-*	2	
	*aadA2*	-*	-*	-*	1	
	*aadA5*	-*	1	-*	-*	
	*aph(3′)-Ia*	-*	-*	-*	3	
	*aph(3″)-Ib*	-*	-*	1	3	
	*aph(6)-ld*	-*	-*	1	3	
Beta-lactam	*blaTEM-1B*	-*	-*	-*	1	5 (6)
	*blaTEM-57*	-*	-*	-*	3	
	*blaCTX-M1*	-*	-*	-*	1	
Folate pathway antagonist	*dfrA12*	-*	-*	-*	1	2 (3)
	*dfrA17*	-*	1	-*	-*	
Phenicol	*floR*	-*	-*	1	5	6 (8)
Macrolide	*mdfA*	8	8	8	8	32 (41)
Quinolone	*qnrB19*	-*	-*	1	3	4 (5)
Sulfonamide	*sul1*	-*	-*	-*	1	6 (8)
	*sul2*	-*	1	-*	3	
	*sul3*	-*	-*	-*	1	
Tetracycline	*tet(A)*	-*	-*	1	5	9 (11)
	*tet(B)*	-*	-*	-*	3	
All AMR genes number (%)		8 (10)	11 (14)	13 (16)	48 (60)	

Abbreviations: TMF, tank milk farm; WMF, waste milk farm. The number and percentage of each resistance gene were analyzed individually in each group. The total number of resistance genes in each group and their percentage of the total identified genes (*n* = 80) is shown. -*, no gene was identified.

**Table 2:** In silico and phenotypic characterization of 32 *E. coli* genomes.

Animal	Sample ID	SRR Accession No.	AMR Gene Profile	Predicted Resistance	AMR Phenotype	Phylogroup	Serotype	ST
TMF Cattle	UPR-0066	SRR10987123	*mdf(A)*	MCL	-*	B1	O150:H8	906
	UPR-0069	SRR10987153	*mdf(A)*	MCL	-*	B1	O117:H9	10783
	UPR-0072	SRR10990262	*mdf(A)*	MCL	-*	B1	OXY60:H21	56
	UPR-0073	SRR10987140	*mdf(A)*	MCL	-*	B1	SB13:H3	4623
	UPR-0074	SRR10987149	*mdf(A)*	MCL	-*	D	O1:H18	38
	UPR-0075	SRR10987248	*mdf(A)*	MCL	-*	A	O66:H25	2325
	UPR-0077	SRR10987249	*mdf(A)*	MCL	-*	E	O158:H9	10761
	UPR-0078	SRR10987152	*mdf(A)*	MCL	-*	B1	O88:H25	5765
TMF Calves	UPR-0079	SRR10987160	*mdf(A)*	MCL	-*	B1	O149:H8	4392
	UPR-0080	SRR10990269	*mdf(A)*	MCL	-*	B2	O40:H30	8217
	UPR-0081	SRR10987150	*aadA5*, *dfrA17*, *mdf(A)*, *sul2*	MCL, SMZ, STM, TMP	STM, SXT	B1	OXY13:H40	155
	UPR-0082	SRR10990363	*mdf(A)*	MCL	-*	B1	O8:H30	8217
	UPR-0083	SRR10987132	*mdf(A)*	MCL	-*	B1	O40:H30	8217
	UPR-0084	SRR10987130	*mdf(A)*	MCL	-*	B1	O8:H30	8217
	UPR-0085	SRR10987119	*mdf(A)*	MCL	-*	A	O177:H11	765
	UPR-0087	SRR10987158	*mdf(A)*	MCL	-*	A	O1:H18	38
WMF Cattle	UPR-0037	SRR10990315	*mdf(A)*	MCL	-*	B1	O90:H7	3580
	UPR-0038	SRR10987121	*mdf(A)*	MCL	-*	B1	O21:H21	101
	UPR-0039	SRR10990265	*mdf(A)*	MCL	-*	B1	O90:H7	3580
	UPR-0042	SRR10987156	*mdf(A)*	MCL	-*	B1	OXY38:H7	10759
	UPR-0043	SRR10990242	*aph(3″)-Ib*, *aph(6)-Id*, *mdf(A)*, *floR*, *qnrB19*, *tet(A)*	CHL, CIP, FLO, MCL, STM, TET	AMC, STM, TET	D	O141:H32	1721
	UPR-0045	SRR10987145	*mdf(A)*	MCL	-*	A	O174:H16	2280
	UPR-0050	SRR10990246	*mdf(A)*	MCL	-*	E	OXY20:H28	942
	UPR-0051	SRR10987139	*mdf(A)*	MCL	-*	B1	O53:H45	10760
WMF Calves	UPR-0053	SRR10987157	*mdf(A)*	MCL	-*	B1	O177:H11	765
	UPR-0054	SRR10987162	*mdf(A)*	MCL	-*	A	O53:H51	5768
	UPR-0055	SRR10987159	*aac(3)-IId*, *aph(3′)-Ia*, *aph(3″)-Ib*, *aph(6)-Id*, *blaCTX-M-1*, *blaTEM-57*, *floR*, *mdf(A)*, *sul2*, *tet(A)*, *tet(B)*	AMC, CHL, ERT, GEN, KAN, MCL, STM, TET	AMP, GEN, KAN, STM, TET	B1	O154:H25	58
	UPR-0058	SRR10987154	*ant(3″)-Ia*, *aph(3′)-Ia*, *floR*, *qnrB19*, *mdf(A)*, *sul3*, *tet(A)*, *tet(B)*	CHL, CIP, KAN, MCL, SMZ, STM, TET	KAN, STM, TET	A	O108:H34	10598
	UPR-0059	SRR10987133	*ant(3″)-Ia*, *aph(3′)-Ia*, *aph(3″)-Ib*, *aph(6)-Id*, *blaCTX-M-1*, *blaTEM-57*, *floR*, *qnrB19*, *mdf(A)*, *sul2*, *sul3*, *tet(A)*, *tet(B)*	AMP, CIP, ERT, KAN, MCL, SMZ, STM, TET	AMP, KAN, STM, TET	A	O108:H35	10598
	UPR-0060	SRR10990264	*mdf(A)*	MCL	-*	A	OXY72:H23	224
	UPR-0061	SRR10987144	*aph(3′)-Ia*, *aph(3″)-Ib*, *aph(6)-Id*, *blaCTX-M-1*, *blaTEM-1B*, *blaTEM-57*, *floR*, *mdf(A)*, *sul2*, *tet(A)*, *tet(B)*	AMP, CHL, CIP, ERT, KAN, MCL, SMZ, STM, TET	AMP, KAN, STM, TET	B1	O38:H26	10758
	UPR-0063	SRR10987120	*aadA2*, *dfrA12*, *floR*, *qnrB19*, *mdf(A)*, *sul1*, *tet(A)*	CHL, CIP, MCL, SMZ, STM, TET, TMP	STM, TET, STX	D	O27:H29	398

Abbreviations: AMP, ampicillin; CHL, chloramphenicol; CIP, ciprofloxacin; ERT, erythromycin; FLO, Florfenicol; GEN, gentamicin; KAN, kanamycin; MCL, macrolide, SMZ, sulfamethoxazole/trimethoprim; ST, Sequence Type; STM, streptomycin; TET, tetracycline; TMF, tank milk farm; WMF, waste milk farm. All sequence data have been submitted to the GenBank database. -* No resistance was observed.

**Table 3:** Overall correlation between AMR phenotype and genotype.

Antibiotic Class	Phenotype Resistant	Genotype Resistant	Phenotype Sensitive	Genotype Sensitive	Correlation (%)
Aminoglycoside	7	7	25	25	100
Beta-lactam	4	5	28	27	96
Macrolide	-*	32	-*	0	-*
Phenicol	-*	32	-*	0	-*
Quinolone	3	0	29	32	91
Sulfonamide	6	2	26	30	87
Tetracycline	6	6	26	26	100

-* Not evaluated phenotypically.

## Data Availability

The sequence reads and assembled genomes were deposited in NCBI under BioProject number PRJNA357722. The SRA accession numbers are SRR10987123, SRR10987153, SRR10990262, SRR10987140, SRR10987149, SRR10987248, SRR10987249, SRR10987152, SRR10987160, SRR10990269, SRR10987150, SRR10990363, SRR10987132, SRR10987130, SRR10987119, SRR10987158, SRR10990315, SRR10987121, SRR10990265, SRR10987156, SRR10990242, SRR10987145, SRR10990246, SRS6078219, SRR10987157, SRR10987162, SRR10987159, SRR10987154, SRR10987133, SRR10990264, SRR10987144, and SRR10987120.
